# S09-3: Play to Prevent: An 8-week coach led FMS for children with intellectual disabilities

**DOI:** 10.1093/eurpub/ckae114.238

**Published:** 2024-09-26

**Authors:** Hayley Kavanagh

**Affiliations:** Sport Ireland, Dublin, Ireland, Dublin city University, Dublin, Ireland, Special Olympics Ireland, Ireland

## Abstract

**Purpose:**

Fundamental movement skills (FMS) represent the ‘building blocks’ of specific movement skills, like jumping and running, and are necessary for engaging in physical activity and sports. Children with intellectual disabilities (CwID) must be provided with opportunities to learn, practice and reinforce these skills.

**Methods:**

A meta-analysis of studies comparing the FMS proficiency of CwID to typically developing children (TDC) (n = 3525, age 4-12 years) showed that the standardised mean difference in FMS performance between these groups is large (g = 1.24). Research investigating FMS proficiency of CwID attending the Special Olympics Ireland (SOI) Young Athletes programme and their typically developing peers (n = 96, 60% aged 5-12 years) demonstrated higher levels of FMS mastery/near mastery amongst TDC. Overall, findings highlighted sub optimal levels of FMS proficiency amongst CwID and the need for tailored FMS interventions targeting specific skill weakness for this population. Currently within the literature, there are no recommended gold standard FMS interventions for improving motor skill proficiency amongst CwID. This study sought to investigate whether a coach led FMS intervention incorporating parental involvement, improves FMS proficiency in CwID attending the SOI YA programme. CwID (n = 90) between 4-12 years participated in the 8-week intervention delivered by coaches (n = 8) in clubs (n = 8) across the island of Ireland. The FMS intervention involved a three-pronged approach, 30 children were in the control group, 30 children in the FMS intervention and 30 children participated in FMS + parent group. Parents of children involved in the third intervention group (n = 30) undertook PLAYshop, a physical literacy workshop designed to upskill parents on the importance of FMS/physical literacy.

**Results:**

Significant improvements were seen in FMS proficiency of children participating in the FMS only and FMS + parent groups. Parents' self-reported knowledge and confidence to support their child's FMS development significantly increased after PLAYshop participation.

**Conclusions:**

Findings support an 8-week, coach led FMS intervention with parental involvement can significantly improve motor skill proficiency of CwID.

**Support/Funding Source:**

Sport Ireland, SOI, Dublin City University.

